# Random *k*-Body Ensembles for Chaos and Thermalization in Isolated Systems

**DOI:** 10.3390/e20070541

**Published:** 2018-07-20

**Authors:** Venkata Krishna Brahmam Kota, Narendra D. Chavda

**Affiliations:** 1 Theoretical Physics Division, Physical Research Laboratory, Ahmedabad 380009, India; 2 Department of Applied Physics, Faculty of Technology & Engineering, The Maharaja Sayajirao University of Baroda, Vadodara 390001, India

**Keywords:** embedded ensembles, *k*-body interactions, lie algebras, fermions, bosons, thermalization, fidelity, *q*-hermite polynomials

## Abstract

Embedded ensembles or random matrix ensembles generated by *k*-body interactions acting in many-particle spaces are now well established to be paradigmatic models for many-body chaos and thermalization in isolated finite quantum (fermion or boson) systems. In this article, briefly discussed are (i) various embedded ensembles with Lie algebraic symmetries for fermion and boson systems and their extensions (for Majorana fermions, with point group symmetries etc.); (ii) results generated by these ensembles for various aspects of chaos, thermalization and statistical relaxation, including the role of *q*-hermite polynomials in *k*-body ensembles; and (iii) analyses of numerical and experimental data for level fluctuations for trapped boson systems and results for statistical relaxation and decoherence in these systems with close relations to results from embedded ensembles.

## 1. Introduction

Random matrix theory (RMT), which is developing constantly into new areas of physics and mathematics, has now became an important part of theoretical physics. As was recognized by Wigner and Dyson very early [1] and has become well established in the last decade, RMT is essential for the description of isolated finite quantum systems and, in particular, quantum chaos, thermalization, decoherence, fidelity decay and many other related aspects that define the statistical mechanics/statistical physics of these systems [2,3,4,5,6]. Investigations into canonical Gaussian orthogonal, unitary and symplectic ensembles (GOE, GUE and GSE) and their various modified versions has been wide spread since early 1980s and their tremendous success is well documented in many books (see, for example, references [5,7,8,9,10,11]) and review articles [12,13,14]. However, one particular class of random matrix ensembles that is now well understood to form paradigmatic models for chaos and thermalization in finite quantum systems (atoms, nuclei, bose gases, mesoscopic systems, such as quantum dots and so on) is the embedded random matrix ensembles (simply embedded ensembles or EE) that are generated by random *k*-body interactions (k=2 being the most important), with or without a mean-field one-body part, and act on many particle Hilbert spaces. With interactions (of low-body rank) and symmetries playing key roles in finite quantum systems, many different EE have been introduced and analyzed, though there are still many unsolved problems, such as the analytical derivation of the two-point correlation function [5,6,15,16,17]. More interestingly, there are many other random matrix ensembles that are closely related to EE, and they will be described in the following text. Focusing on thermalization, many concepts, such as quantum chaos, de-localization transitions, the eigenstate thermalization hypothesis (ETH), spreading of an eigenstate over an energy shell, fidelity decay, the emergence of Gibbs ensemble, canonical typicality and so on have been studied using EE in some selective investigations in the last decade [6,18,19,20,21,22,23,24]. In addition, with recent developments in carrying out controlled experiments with cold atoms, new theoretical studies of trapped boson systems applying RMT have emerged [25,26,27,28,29]. The purpose of the present article is to give a brief review of these developments with an emphasis on new results. Though there was a recent review on embedded ensembles [6], the overlap of the present article with that review is kept to a minimum. The focus in the previous review is on nuclear structure applications of EE. Now, we give a preview.

In Section 2, briefly discussed are different types of EE, based on symmetries (Lie algebras, point groups and so on) and Bose or Fermi (or Majorana) characteristics and so on. In Section 3, basic results of EE(1+k) for thermalization in finite quantum systems are presented. Section 4 gives some applications of RMT to atomic trapped boson systems. Finally, Section 5 presents the conclusions.

## 2. Random Matrix Ensembles with Symmetries for Fermi, Bose and Other Systems

Finite isolated quantum systems, such as atoms, nuclei, mesoscopic devices of condensed matter, spin systems modeling a quantum computing core and so on, have interactions among their constituents which play a vital role in determining their properties. Therefore, the random matrix ensembles incorporating this feature as well as the nature of the constituents (fermions, bosons, Majorana fermions and so on) and symmetries carried by the interactions are being studied in detail. These ensembles are, in general, called EE. In the subsections to follow, we will briefly discuss these ensembles.

### 2.1. Embedded Ensembles for Fermi and Bose Systems with Lie Symmetries

#### 2.1.1. Ensembles for Fermion Systems

Given a system of *m* spinless fermions in *N* degenerate single particle (sp) states, and say the interaction among the fermions is a *k*-body interaction (k≤m), the Hamiltonian operator, *H*, for the system takes the form

(1) $$H=V\left(k\right)=\sum_{k_{a},k_{b}}V_{k_{a},k_{b}}A^{†}\left(k_{a}\right)A\left(k_{b}\right).$$

Here, $A^{†}\left(k_{a}\right)$ creates a normalized *k* particle state, $\left|k_{a}\right.\rangle$, with $A^{†}\left(k_{a}\right)\left|0\right.\rangle =\left|k_{a}\right.\rangle$. Similarly, $A(k_{b})$ is a *k* particle annihilation operator. Note that $V_{k_{a},k_{b}}$ are the matrix elements of *H* in the *k* particle (defining) space, with the *V* matrix dimension being dk=Nk. Now, representing the *V* matrix by GOE, we have a GOE ensemble of *H* operators and the actions of each member of this GOE on the *m* particle states will generate an *m* particle *H* matrix of dimensions dm=Nm. The ensemble of these matrices form an embedded GOE of *k* particle interactions (FEGOE(*k*) with *F* for fermions) in *m* particle spaces. Similarly, GUE representation of the *V* matrix will generate FEGUE(*k*) in *m* particle spaces. Although GSE embedding is also possible, we do not consider this in the remainder of this article. As the Hamiltonian given by Equation (1) does not include spin or other internal degrees of freedom for the fermions, the FEGOE(*k*) and FEGUE(*k*) are for spinless fermion systems. The GOE/GUE structure in *k* particle spaces is propagated to *m* particle spaces by U(N) algebra with U(N) generated by $a_{i}^{†}a_{j}$, where *i* and *j* are sp states. Note that, for example, FEGUE(*k*) is invariant under $U(N_{k})$ action in *k* particle spaces but not under $U(N_{m})$. Similarly, a GUE in *m* particle spaces is invariant under $U(N_{m})$. These ensembles have been analyzed by many research groups (see, for example, references [5,6,15,16,30] and references therein). An important aspect of these ensembles is that all their properties follow from the Wigner–Racah algebra of the embedding U(N) algebra [5].

Going further, it is possible to incorporate internal degrees of freedom, such as spin, for fermion systems in fermionic embedded ensembles (FEE). In general, it is possible to construct FEE with U(N)⊃U(N/r)×SU(r) embedding algebra with *H* being a SU(r) scalar. Note that the *m* particle space divides now into subspaces labeled by SU(N/r) irreducible representations (irreps) that are, in turn, labeled by Young tableaux, $\left\{f_{1},f_{2},\ldots ,f_{N/r}\right\}$, with $\sum_{i}f_{i}=m$ and $f_{1}\geq f_{2}\geq \ldots \geq f_{N/r}\geq 0$. Also, the U(r) irreps (they can be easily converted into SU(r) irreps) are conjugated to the U(N/r) irreps. These FEE are studied for r=2 which corresponds to the spin (*s*), and these ensembles are important, for example, in atoms and quantum dots. Similarly, r=4 corresponds to the spin-isospin, SU(4), used in atomic nuclei. More specifically, the r=2,4 ensembles (note that r=1 gives trivially FEGOE(*k*) and FEGUE(k)) are analyzed in some detail for two-body (k=2) interactions, and it is possible to extend this for k≥3. Note that for k=2, the *H* divides into two parts that transform into {2} and $\left\{1^{2}\right\}$ irreps with respect to U(r). In addition, for fermion systems, EE with parity symmetry, $U\left(N_{1}+N_{2}\right)⊃U\left(N_{1}\right)⊕U\left(N_{2}\right)$ symmetry for two types of fermions with fermion numbers preserved, U(N)⊃SO(3) with SO(3) generating angular momentum *J* and U(2N)⊃SO(3)⊗SU(2) with SU(2) generating isospin, are also developed and applied. Besides *k*-body ensembles, it is also possible to consider ensembles with a mixed body rank. Most important among these is EE with fermions in a mean-field (generated by a one-body operator $h\left(1\right)=\sum_{i}\epsilon_{i}n_{i}$ with $\epsilon_{i}$ being the sp energies and $n_{i}$ being the number operator for the *i*th sp state) and interacting with a *k*-body interaction. Then, we have FEE(1+k) with

(2) H=h(1)+λV(k),

where λ is the strength of the *k*-body interaction measured in units of the average mean spacing of the sp energies defining h(1). A very significant property of both FEE(*k*) and FEE(1+k) is that, in general, they exhibit a Gaussian to semi-circle transition in the eigenvalue density as *k* changes from a low *k* value to *m*. This is discussed further in Section 3 with application to some aspects of thermalization.

#### 2.1.2. Ensembles for Boson Systems

**Figure 1:**
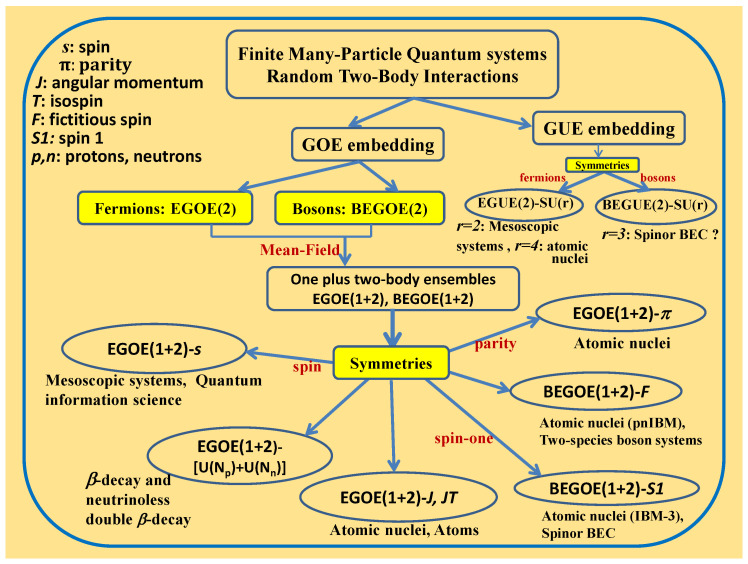
Lie algebraic embedded ensembles and some of the quantum systems where they are applicable. See [5] for details and the figure is taken from this reference with permission from Springer.

All the ensembles discussed above extend directly to interacting boson systems with, say, *m* bosons in *N* sp states. Then, firstly, we obtain BEGOE(*k*) and BEGUE(*k*), for spinless bosons, defined by the Hamiltonian in Equation (1) by replacing creation and annihilation operators by those for bosons with proper normalization. Note that Nk=N+k−1k, Nm=N+m−1m, and *B* stands for bosons. Let us add that there is an “uncomfortable” case of many bosons distributed in two single-particle levels with random *k*-body interactions, which has been proven to be non-ergodic and hence, no thermalization should be expected here [31]. This is an important result as this case corresponds to a two-mode Bose–Einstein condensation (BEC) which has been experimentally realized [32]. Going further, it is also easy to define BEGOE(1+k) and BEGUE(1+k) using Equation (2). More importantly, it is possible to consider bosonic embedded ensembles (BEE) with U(N)⊃U(N/r)×SU(r), and clear applications of these with r=2 and 3 are for two-species BEC and spinor BEC, respectively. However, for bosons, the U(r) and U(N/r) irreps are the same. See Figure 1 and [5] and references therein for details of FEE and BEE with Lie algebra symmetries. Going further, it is possible to construct ensembles for Bose–Fermi (BF) systems based on the interacting boson–fermion model (IBFM) of atomic nuclei (see references [33,34,35,36,37] for IBFM Hamiltonians with one, two and three fermions coupled to bosons). Treating the parameters in these *H*s as Gaussian variables will generate EE for BF systems. It will be interesting to investigate this class of ensembles in future, as the ensembles with one and three fermions are applicable for example to odd-mass atomic nuclei [33]. It is also possible to incorporate Lie algebraic symmetries into BF embedded ensembles.

### 2.2. Embedded Ensembles with Point Group Symmetries and Others

In addition to FEE or BEE with Lie algebraic symmetries, it is interesting to note that it is possible to consider point group symmetries. These are of obvious importance for molecules and clustering in atomic nuclei [38,39]. We illustrate this with the example of the quantal motion of a system of four identical particles with tetrahedral symmetry ($T_{d}$). The relative motion of the four-particle system with, say, its co-ordinates, $\vec{r_{1}}$, $\vec{r_{2}}$, $\vec{r_{3}}$ and $\vec{r_{4}}$, is described by the three Jacobi co-ordinates, $\vec{\rho }=\left(\vec{r_{1}}-\vec{r_{2}}\right)/\sqrt{2}$, $\vec{\lambda }=\left(\vec{r_{1}}+\vec{r_{2}}-2\vec{r_{3}}\right)/\sqrt{6}$, $\vec{\eta }=\left(\vec{r_{1}}+\vec{r_{2}}+\vec{r_{3}}-3\vec{r_{4}}\right)/\sqrt{12}$, and their conjugate momenta. Bosonic quantization of this system by adding a scalar (*s*) boson gives rise to a four-level interacting boson model with three levels carrying angular momentum, $1^{-}$ (they correspond to ρ, λ and η bosons), and one with angular momentum, $0^{+}$ (corresponds to *s* boson). Then, the boson creation operators are $s^{†}$, $b_{\rho }^{†}$, $b_{\lambda }^{†}$ and $b_{\eta }^{†}$; these can be labeled, for convenience, as bosons 1, 2, 3 and 4 respectively. Using these and the corresponding annihilation operators, it is easy to write a general one plus two-body Hamiltonian that preserves the total boson number, $n=n_{s}+n_{\rho }+n_{\lambda }+n_{\eta }$, and total angular momentum, *L*. Then, we have U(10)⊃SO(3)⊃SO(2) algebra with U(10) irreps being totally symmetric (for *N* bosons, the irrep is {N}). Now, it is possible to impose $S_{4}$ (permutation group of 4 objects) or $T_{d}$ symmetry on the Hamiltonian. To this end, one may recognize that the basic permutations of four objects are the transposition, P(12), and cyclic permutation, P(1234), and all other permutations follow from these. Also, with respect to $S_{4}$, the many-boson space divides into five irreps, $\left\{4\right\}∼A_{1}$, $\left\{31\right\}∼F_{2}$, {22}∼E, $\left\{211\right\}∼F_{1}$ and $\left\{1111\right\}∼A_{2}$. Using the actions of P(12) and P(1234) on the four creation and annihilation operators, one can write one-body operators with definite $S_{4}$ irrep character and therefore, a one plus two-body Hamiltonian with $S_{4}$ symmetry. See Equation (12) of reference [39] for the explicit form of this *H* which contains 12 free parameters. Choosing these as independent Gaussian random variables generates a BEE with $S_{4}$ symmetry. By diagonalizing the P(12) and P(1234) operators in the boson space, it is possible to identify the EE that belong to a given $S_{4}$ irrep. Numerical and analytical studies of BEE with point group symmetries will be interesting and may prove to be useful.

Besides point group symmetries, it is indeed possible to consider the matrix in the defining space of the interactions of a quantum system to have a specific structure. For example, one may consider centrosymmetric matrices, circulant matrices, the more general Toeplitz matrices and so on. An N×N matrix is said to be centrosymmetric if it commutes with the exchange matrix, *J*, where $J_{i,j}=\delta_{i,N-j+1}$. Similarly, a Toeplitz matrix is defined by $A_{i,j}=A_{i+1,j+1}=a_{i-j}$. A circulant matrix, A, is a Toeplitz matrix with an additional condition, $a_{i}=a_{i+n}$. Also, a symmetric Toeplitz matrix is centrosymmetric. One important issue here is that these matrix structures may not be preserved by propagating the interaction matrix to many particle spaces. For example, for a *k*-body interaction, the *H* matrix in the *k* particle space may be chosen to be centrosymmetric, but then, in the *m* particle spaces, the centrosymmetry will not be, in general, preserved. In the specific example of centrosymmetric matrices, Benet et al imposed the symmetry at the one particle level and then constructed FEGOE(*k*), FEGUE(*k*), BEGOE(*k*) and BEGUE(*k*) ensembles. They showed that EEs with centrosymmetry enhance the transport efficiency in quantum systems [40,41].

In addition to GOE, GUE and GSE classical ensembles (they are based on time-reversal and rotational invariance), chiral symmetry gives three more ensembles [42], and similarly, charge conjugation symmetry gives four more ensembles [43]. The embedding of these seven ensembles in addition to GOE, GUE and GSE embeddings will generate new classes of EE. Constructing and analyzing these EEs may prove to be useful in the future.

### 2.3. Ensembles for Majorana Fermions and Other Systems

Recently, it was recognized that the late time behavior of horizon fluctuations in large anti-de Sitter (AdS) black holes is governed by random matrix dynamics, and the main tool used here is the so called Sachdev–Ye–Kitaev (SYK) model [44]. A remarkable property of the SYK model is that the zero temperature entropy scales with the number of particles. See reference [45,46] for a detailed discussion on this. The SYK model Hamiltonian for *N* strongly interacting Majorana fermions with infinite-range 4-body interactions takes the form

(3) $$H=\frac{1}{4!}\sum_{i,j,k,l=1}^{N}X_{ijkl}\chi_{i}\chi_{j}\chi_{k}\chi_{l}\;;\;\;\left\{\chi_{i},\chi_{j}\right\}=2\delta_{ij}\;.$$

Here, $\chi_{i}$ are Majorana fermions, and their algebra is same as that of the Dirac γ matrices. Note that the *H* matrix dimension is $2^{N/2}$. Choosing $X_{ijkl}$ to be independent Gaussian random variables, we have a random matrix ensemble for *H*. We call this the Majorana random matrix ensemble (MRE). It is easy to see from Equation (3) that the MRE is similar to EE. This is further confirmed by reference [47] where it is shown that the (central) moments, $M_{2p}$, of the eigenvalue density generated by *H* for large, but finite, *N* and *k*-body interactions are given by

(4) M2p(M2)p=(1−η)p∑r=−pr=p(−1)r2pp+rηp(p−1)/2;η=Nk−1∑r=0k(−1)k+rkrN−kk−r.

These are, indeed, the moments of the generating function of the so-called *q*-hermite polynomials with q=η. Their explicit forms and some other details are given ahead in Section 3.2. Let us add that this generating function changes from Gaussian for q=η=1 to semi-circle for q=η=0 [48]. Thus, the eigenvalue density for the SYK model, *H*, i.e., for MRE, is intermediate to Gaussian and semi-circle, and this has been well verified by numerical calculations. For N≫1 and *E* values not close to the edge of the spectrum, the approximate expression for the eigenvalue density is $\rho_{asmp}\left(E\right)=c_{0}\;\exp\left(2\arcsin^{2}\left(E/E_{0}\right)/\log\eta \right)$, where η is given by Equation (4) and $E_{0}$ is the ground state energy per particle [47]. It is important to note that for *m* fermion or boson system, the eigenvalue density generated by EE(*k*) changes from Gaussian to semi-circle as *k* changes from 1 to *m* (see Section 3.2 ahead). It is important to add that the $\rho_{asmp}\left(E\right)$ generated by the SYK model shows exponential growth in level density for energies close to the ground state, just like the well known Bethe’s [49] level density formula that applies to complex nuclei. Going beyond the eigenvalue density, also studied are the distribution of the lowest eigenvalues, level statistics and the partition function generated by MRE [47]. Without going into detail, it is important to add that MRE can be visualized in terms of EE(*k*), and many results for MRE follow from those of EE(*k*) (see, for example, reference [47,50]).

Another class of models that is close to EE is the Hamiltonians used for spin chains or spin networks, such as tight-binding Hamiltonians. Although there is no explicit embedding of a *k*-body interaction in *m*-particle spaces in these systems, a few-body character for interactions is used in these models [51]. It has been seen in the past [4] that many results of interacting spin models are similar to those given by EE(*k*) and EE(1+k). More recent and interesting work in this direction is the demonstration that quantum spin glasses on arbitrary graphs show a transition in the density of states from Gaussian to semi-circle [52], just as in EE(*k*). It is interesting and important to explore further the equivalence in generating statistical properties between various interacting spin models and EE.

## 3. Delocalization, Quench Dynamics and Thermalization in EE

To understand the thermalization of isolated finite quantum systems with the constituents in a mean-field and interactions with low-body rank interactions, EE(1+k) with *H*, given by Equations (1) and (2), can be used as a generic model. In the next three subsections, we discuss the results from this random matrix model.

### 3.1. Localization–Delocalization Transitions and Thermalization

Let us first consider EGOE(1+k) with k=2, i.e., fermions or bosons in a mean-field with two-body interactions giving {H}=h(1)+λ{V(2)} with V(2), a GOE in two-particle spaces; {} denotes an ensemble. Without any loss in generality, we assume that the average mean spacing of the sp energies defining h(1) is, say, Δ, and the interaction strength, λ, is measured in units of Δ.

Firstly, EGOE(1+2) generates Gaussian eigenvalue densities independent of λ (i.e., the shape of the eigenvalue density is independent of λ), and the convergence to Gaussian is asymptotic. For small λ, there will be Poisson fluctuations, and therefore, the smoothed Gaussian form will have larger fluctuations. As λ increases from a value of zero, after crossing a value, $\lambda_{c}$, there will be a transition (truly, a cross-over) in level fluctuations from Poisson like to Wigner–Dyson (i.e., GOE). In EGOE(1+2), just as in many realistic systems, the behavior of various observables continues to evolve, even after the GOE nature of the level fluctuations has stabilized, with the strength, λ, of the perturbation. Therefore, more generally, rather than level statistics, the (chaotic) structure of eigenstates defines quantum chaos. At this stage, it is important to recognize that for very small λ values, all eigenstates are localized in the Fock space of the h(1) states (basis states). As λ increases, the eigenstates start spreading and the basic ingredients’ strength functions take the Breit–Wigner (BW) form. Given the h(1) states, $\left|k\right.\rangle$, and eigenstates, $\left|E\right.\rangle$, with $\left|E\right.\rangle =\sum_{k}C_{k}^{E}\left|k\right.\rangle$, the strength functions, $F_{k}\left(E\right)$, are defined by

(5) $$F_{k}\left(E\right)=\sum_{E^{\prime }}{|C_{k}^{E^{\prime }}|}^{2}\;\delta \left(E-E^{\prime }\right)=d\;\bar{{\left|C_{k}^{E}\right|}^{2}}\;\rho \left(E\right)\;,$$

where $\bar{|C_{k}^{E}{|}^{2}}$ is the average of $|C_{k}^{E}{|}^{2}$ over the degenerate *E* states; *d* is the dimension of the *m*-particle space; and ρ(E) is the normalized eigenvalue density.

**Figure 2:**
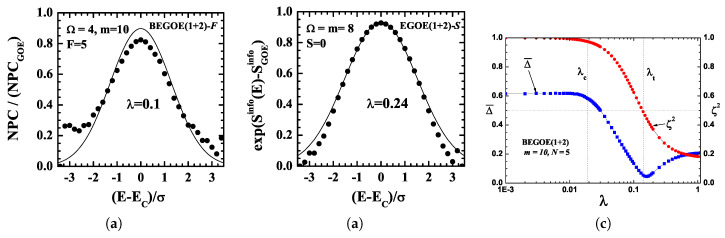
(**a**) Example of the number of principal components (NPC) vs. the normalized energy, $\hat{E}=\left(E-E_{c}\right)/\sigma$, for a bosonic BEGOE(1+2)-*F* ensemble with m=10 bosons in Ω=4 sp orbits, each doubly degenerate with a fictitious spin, F=5. The numerical ensemble averaged results are represented by filled circles, while the continuous curve is from the formula for NPC (for further detail, see reference [20]). Note that the NPC value for GOE is 1 in the graph. The result clearly shows dynamical localization and very strong deviation from GOE. This implies that there is a spread of the eigenstates over a energy shell and not over the entire basis of states. (**b**) $S^{info}$ vs. $\hat{E}$ for a fermionic EGOE(1+2)-*s* ensemble for m=8 fermions in Ω=8 sp orbits, each doubly degenerate with spin S=0. The numerical ensemble averaged results are represented by filled circles, while the continuous curve is from the formula for $S^{info}$ (for further detail, see reference [19]). (**c**) Example showing a region of thermalization generated by EE as a function of the two-body interaction strength λ. Results are shown for a spinless BEGOE(1+2) ensemble with m=10 bosons in N=5 sp states. In the figure, $\bar{\Delta }={\left\{\int_{-\infty }^{\infty }\left[{\left(R_{E}^{info}-R_{E}^{ther}\right)}^{2}+{\left(R_{E}^{sp}-R_{E}^{ther}\right)}^{2}\right]dE\right\}}^{1/2}/\left\{\int_{-\infty }^{\infty }R_{E}^{ther}dE\right\}$, where $R_{E}^{\alpha }=\exp(S^{\alpha }\left(E\right)-S_{max}^{\alpha })$. For the definition of the thermodynamic entropy and sp entropy and all other details, see reference [54]. Note that $\bar{\Delta }∼0$ implies that all entropies are the same, and therefore, this defines the region of thermalization. In the figure, this happens for $\lambda =\lambda_{t}$. Similarly, $\zeta^{2}$ is the correlation coefficient given by $\zeta^{2}=\frac{\sigma_{h(1)}^{2}}{\sigma_{h(1)}^{2}+\lambda^{2}\sigma_{V(2)}^{2}}$. The $\lambda_{t}$ marker for the thermodynamic region corresponds to $\zeta^{2}=0.5$. The $\lambda_{c}$ marker is also shown in the figure.

More significantly, as λ increases beyond $\lambda_{c}$, strength functions change (at $\lambda =\lambda_{F}>\lambda_{c}$) from the BW to the Gaussian form, and with further increase, there will be thermalization with maximal wavefunction delocalization within an energy shell, i.e., the eigenstates will spread over many basis states but not over all basis states. This is clearly seen from the results, as shown in Figure 2, for the chaos measure number of principal components (NPC or ξ) and the information entropy ($S^{info}$) both are defined in terms of $|C_{k}^{E}{|}^{2}$,

(6) $$\xi \left(E\right)={\left\{\sum_{k}{\left|C_{k}^{E}\right|}^{4}\right\}}^{-1}\;,\;\;S^{info}\left(E\right)=-\sum_{k}{\left|C_{k}^{E}\right|}^{2}\;\ln{\left|C_{k}^{E}\right|}^{2}\;.$$

In the thermalization region, $\lambda ∼\lambda_{t}$, and the spreading produced by h(1) and V(2) will be equal, generating maximum mixing with Gaussian strength functions and GOE fluctuations. These results have been well verified numerically (also the parametric forms of $\lambda_{c}$, $\lambda_{F}$ and $\lambda_{t}$ are well understood) using EGOE(1+2) and BEGOE(1+2) for spinless fermion and boson systems and also for fermion and boson systems with the spin, $\frac{1}{2}$, degree of freedom (see [5,20] for details). Figure 2 shows some numerical results from EE, and it can be seen from these results that in isolated finite systems, interactions act as the heat bath [4], and there is the phenomena of localized thermal states. The later aspect was investigated in more detail recently in reference [53].

**Figure 3:**
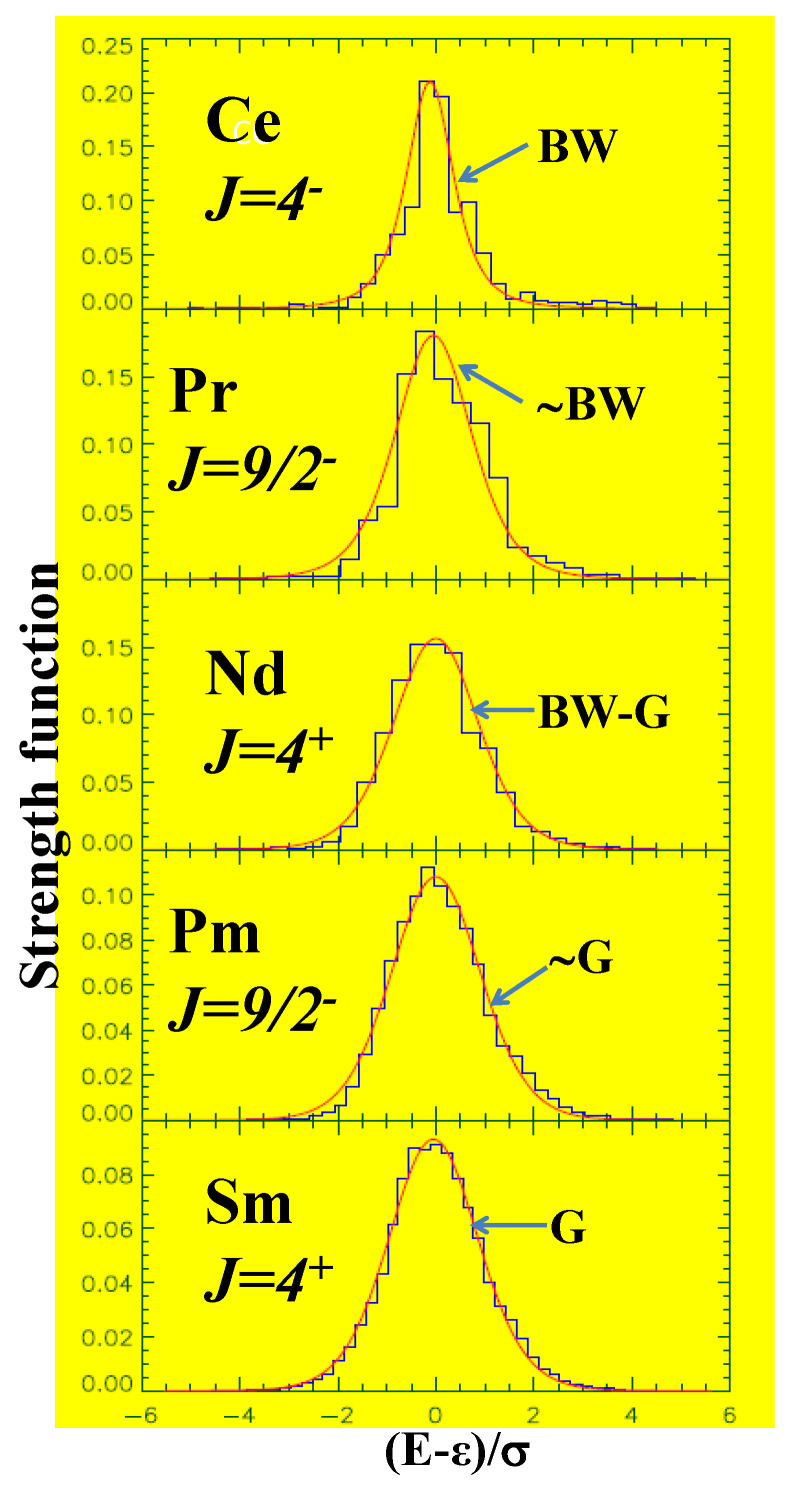
BW to Gaussian transition in strength functions in atoms. Note that CeI is very close to BW, and SmI to Gaussian (denoted by G in the figure), and NdI is intermediate to BW and Gaussian. In addition, PrI is closer to BW and PmI to Gaussian. The histograms are the result of atomic structure calculations and the smooth curve is from a function that interpolates BW and Gaussian. The figure is taken from reference [58] with permission from the American Physical Society.

A large number of numerical studies have shown that complex atomic nuclei lie in the thermalization region ($\lambda ∼\lambda_{t}$); Wigner, Dyson and French, in some way, conjectured this in introducing RMT and EE [1,15]. Exploiting this, methods have been developed to calculate observables that are intrinsically statistical, such as level densities, orbit occupancies, transition strength distributions and so on, and to analyze data (see references [6,55] and references therein). On the other hand, Chirikov recognized, for the first time, using a small spectroscopic space for CeI, that complex atoms are also quantum chaotic [56]. However, detailed study of CeI, PrI, NdI, PmI and SmI showed that atoms exhibit the BW to Gaussian transition in strength function with CeI close to BW and SmI to Gaussian. See Figure 3 and reference [57,58] for detail. Exploiting this, a theory for calculating statistical quantities for atoms was developed by Flambaum et al. with some applications [59]. It is also possible to use this approach to derive bounds on *T*-odd, *P*-even interactions in rare-earth atoms that are chaotic [60].

### 3.2. Relaxation Dynamics Following an Interaction Quench

Going beyond the results of the previous subsection, for further insight into thermalization and also controlled experiments with cold atoms, ion traps, etc. [61,62,63,64,65], the relaxation dynamics of an isolated finite quantum system after a random interaction quench is studied using EE(1+k). Here, strength functions play a major role, and, for *H* given by Equation (2), with a sufficiently large value of λ (i.e., $\lambda ∼\lambda_{t}$), the forms of the strength functions follow those of the eigenvalue densities (with variances and centroids being different). Also, in this situation, the form of the eigenvalue density for EE(1+k) will be same as that of EE(*k*). Therefore, first, we will briefly consider the eigenvalue density for EE(*k*) as *k* changes from 1 to *m*.

#### 3.2.1. Eigenvalue Density for EE(*k*)

Soon after the introduction of EE(*k*), it was recognized that one of the characteristic properties of these ensembles is that they exhibit a Gaussian to semi-circle transition as *k* changes from 1 to *m*. There are numerical results for this from a large number of groups and analytical proofs via lower order moments. However, only now, with the developments in the context of random matrix features of the SYK model described in Section 2.3, the possibility of identifying the analytical form of the eigenvalue density valid for any *k* has arisen.

In mathematics literature, it is well known that *q*-Hermite polynomials are orthogonal with respect to a function v(x|q) that takes Gaussian form for q=1 and semi-circle form for q=0 [48]. Therefore, it is plausible that v(x|q) represents the eigenvalue density of EE (*k*), and this is established by deriving formulas for *q* as a function of (N,m,k) for FEGUE(*k*) and BEGUE(*k*), and they are valid also for the corresponding GOE versions. Let us first introduce *q* numbers, ${\left[n\right]}_{q}$, where ${\left[n\right]}_{q}={\left(1-q\right)}^{-1}\left(1-q^{n}\right)$. Then, ${\left[n\right]}_{q\to 1}=n$, and ${\left[n\right]}_{q}!=\Pi_{j=1}^{n}{\left[j\right]}_{q}$ with ${\left[0\right]}_{q}!=1$. Now, *q*-Hermite polynomials, $H_{n}\left(x|q\right)$, are defined by the recursion relation, [48], $x\;H_{n}\left(x|q\right)=H_{n+1}\left(x|q\right)+{\left[n\right]}_{q}\;H_{n-1}\left(x|q\right)$, with $H_{0}\left(x|q\right)=1$ and $H_{-1}\left(x|q\right)=0$. Note that for q=1, the *q*-Hermite polynomials reduce to normal Hermite polynomials (related to Gaussian) and for q=0, they reduce to Chebyshev polynomials (related to semi-circle). More importantly, *q*-Hermite polynomials are orthogonal within the limits $\pm 2/\sqrt{1-q}$ with the weight function v(x|q) where [48]

(7) $$\begin{array}{ccc} v(x|q) & = & N_{q}\;\sqrt{1-\frac{x^{2}}{x_{0}^{2}}}\;\prod_{\kappa =1}^{\infty }\left[1-\frac{4(x^{2}/x_{0}^{2})}{2+q^{\kappa }+q^{-\kappa }}\right]\;;\;\;\\ x_{0}^{2} & = & \frac{4}{1-q}\;. \end{array}$$

Here, *x* is standardized variable (centroid zero and variance unity); $-2/\sqrt{1-q}\leq x\leq 2/\sqrt{1-q}$; and $N_{q}$ is the normalization constant. It can be seen that in the q→1 limit, v(x|q) will take Gaussian form and in the q=0 limit, semi-circle. Thus, v(x|q) interpolates the Gaussian and semi-circle forms. Lower, even-order, reduced central moments of v(x|q) follow from Riordan and Touchard [66,67] and they are as follows (all odd moments vanish):

(8) $$\begin{array}{c} \mu_{4}\left(q\right)=2+q\;,\\ \mu_{6}\left(q\right)=5+6q+3q^{2}+q^{3}\;,\\ \mu_{8}\left(q\right)=14+28q+28q^{2}+20q^{3}+10q^{4}+4q^{5}+q^{6}\;. \end{array}$$

Now, turning to EE(*k*) and using the formulas derived in the past for $\mu_{4}$, $\mu_{6}$ and $\mu_{8}$ for FEGUE(k) and FEGOE(*k*) using the so-called binary correlation approximation, as given in [30] and [15], respectively, and comparing them with Equation (8), it is established that $q∼\mu_{4}-2$ for these ensembles, and this also extends to BEGUE(*k*) and BEGOE(*k*). Moreover, using the results in [16,68,69] the formulas for *q* as a function of (N,m,k) are obtained for all the above four ensembles in [70]. For example, for BEGUE(*k*) (also valid for BEGOE(*k*)), the formula is

(9) q∼N+m−1m−1∑ν=0νmaxX(N,m,k,ν)dB(gν)ΛB0(N,m,k)2X(N,m,k,ν)=ΛBν(N,m,m−k)ΛBν(N,m,k);ΛBν(N,m,r)=m−νrN+m+ν−1r,dB(gν)=N+ν−1ν2−N+ν−2ν−12.

**Figure 4:**
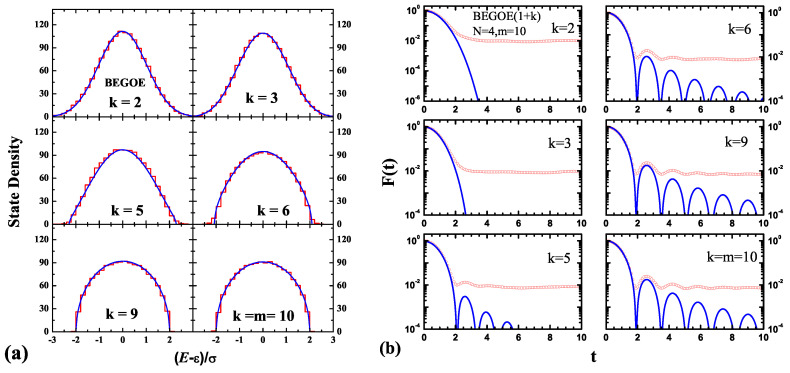
(**a**) The histograms represent the ensemble averaged spectral density of a 100-member BEGOE(*k*) with m=10 bosons in N=4 sp states for different *k* values. The blue solid lines were obtained using Equation (7) with *q* given by Equation (9). (**b**) The fidelity decay, F(t), as a function of time for a 100-member BEGOE(1+k) ensemble with N=4 and m=10 represented by the red open circles; the ψ(0) here corresponds to the middle states of the h(1) spectrum. The blue smooth curves are obtained by taking a numerical Fourier transform of the strength functions represented by Equation (7). Figures are by Priyanka Rao and one of the authors (NDC) [71].

Numerical tests confirmed that v(x|q), given by Equation (7), along with the (N,m,k) dependent formulas for *q*, as given, for example, by Equation (9), describe the eigenvalue density for the four EE(*k*) ensembles. See Figure 4a for an example [70,71].

#### 3.2.2. Survival Probability after an Interaction Quench

The fidelity decay or survival probability of a quantum system after a sudden quench is an important quantity in the study of relaxation of a complex (chaotic) system to a equilibrium state. Say the system is prepared in one of the eigenstates (ψ(0)) of the unperturbed Hamiltonian, $H=H_{0}$, and then with a sudden interaction quench, $H\to H_{0}+V$, the system evolves unitarily with respect to $H=H_{0}+V$, giving ψ(t)=exp(−iHt)ψ(0). Now, the fidelity decay or survival probability, $F\left(t\right)=|\langle \psi (t)|\psi (0)\rangle {|}^{2}$, is the probability of finding the system in its initial, unperturbed state after a given time, *t*. Within RMT, we can replace $H_{0}$ with h(1) and *V* with λV(k) so that the final *H* is same as in Equation (2); thus, we are studying F(t) using EE (1+k). The most significant result that can be derived easily is that F(t) is the Fourier transform of the strength function given by Equation (5); this is valid for times that are not very short or very long. For $\lambda ∼\lambda_{t}$, as already discussed before, we have a Gaussian form for the strength functions for k=2, and it will be semi-circle for k=m. These two extreme situations were studied in detail recently both analytically and numerically in references [21] and [72,73,74]. Without going into detail, let us mention that for *t* that are not too small or very large, the formulas for F(t) are

(10) $$F\left(t\right)\overset{k=2}{⟶}\exp\left(-\sigma_{0}^{2}\;t^{2}\right)\;,\;\;F\left(t\right)\overset{k=m}{⟶}\frac{{\left[J_{1}\left(2\sigma_{0}\;t\right)\right]}^{2}}{\sigma_{0}^{2}\;t^{2}}\;.$$

Here, $J_{1}$ is the Bessel function of the first-kind, and $\sigma_{0}^{2}$ is the variance in λV(k). Clearly, following the results of the previous subsection, v(x|q) can be used for F(t) generated by EE(1+k) for any *k* value. So far, the analytical formula for the Fourier transform of v(x|q) is not available, and hence, it is evaluated numerically. Some examples are shown in Figure 4b, and they demonstrate that v(x|q) describes the survival probability for any *k*, and this bridges the gap between the results reported in reference [21] for k=2 and in reference [72] for k=m. Let us add that fidelity decay is correlated with the entropy generation and its saturation over time [21,72,73,74], i.e., statistical relaxation (See Figure 8a ahead). For any *k*, this can be investigated using v(x|q) and this is a topic for future research.

In the results presented in Figure 4b, the interaction strength, λ, is sufficiently large so that the strength functions exhibit a transition from Gaussian to semi-circle as *k* changes. However, as λ decreases, strength functions take the BW form for fixed *k*. Therefore, by changing both λ and *k*, it is possible to observe the BW to Gaussian to semi-circle transition in strength function. Also, it is possible to have a shape intermediate to BW and semi-circle for some values of λ and *k* [75]. With these, fidelity decay will have more a complex structure with both λ and *k* varying in EE(1+k). It is clearly of interest to explore this both numerically and analytically.

### 3.3. ETH and Ergodicity

Von Neumann, who addressed quantum ergodicity, noted, already, in 1929, that when discussing thermalization in isolated quantum systems, one should focus on the “physical observables” as opposed to the “wave functions or density matrices” describing the entire system [76]. Therefore, although the results of the previous subsections are important, the breakthrough in the topic of thermalization in isolated many-body systems was the eigenstate thermalization hypothesis (ETH) [77,78]. Related to quantum chaos and operation of RMT in many-body systems, ETH states that the eigenstates of generic quantum Hamiltonians (or quantum many-body systems) are “typical” in the sense that the expectation values of observables in these states are the same as those predicted by the microcanonical ensemble. This implies that the expectation values of observables in isolated quantum systems far from equilibrium relax to (almost) time-independent results that can be described using an appropriate statistical (Gibbs) ensemble [77,78,79]. This quantum ergodicity has been verified in several quantum lattice systems [2,4] and also by EE(*k*) [22,24]. We now briefly discuss the results from EE(*k*) testing quantum ergodicity, or ETH.

Starting with a general EE(1) for a system of *m* fermions in *N* sp states with *H*, given by

(11) $$H=\alpha \;\hat{n}+\eta \sum_{i,j}\epsilon_{ij}a_{i}^{†}a_{j}$$

and representing the $\epsilon_{ij}$ matrix by GOE, it was argued by Magan [24] that this system of random free fermions obey ETH for m≫1. Note that in Equation (11), $\hat{n}$ is the number operator (its eigenvalue is *m*) and the matrix of *H* in one-particle space is no longer chosen to be diagonal as in Equation (2). In reference [24], studied analytically, using *H* defined by Equation (11) and with an EE(1) ensemble average in *m*-particle spaces were (i) the energy average and variance; (ii) the average and correlations of $C_{ij}=a_{i}^{†}a_{j}$ matrix; and (iii) the entanglement entropies. It is seen that the leading term in the 1/m expansion of these gives a thermal result with the effective temperature being a function of (N,m). These results, though interesting, in many realistic systems, such as atomic nuclei, atoms etc., it is necessary to examine, using EE(1+2), the ergodicity principle (or ETH) which is the corner stone of thermalization, and we turn to this now.

In reference [22], a first attempt was made to study the relevance of various factors leading to the thermalization of an isolated finite quantum system using EGOE(1+2), and some of these are the structure of the initial state, the nature of spectral fluctuations and delocalization of the wavefunctions in an energy region, the nature of the observables, the dimensions of the Hilbert space and so on. Let us say $\left|\Psi (0)\right.\rangle$ is the initial state of a system so that $\left|\Psi (0)\right.\rangle =\sum_{\mu }C_{\mu }\left|E_{\mu }\right.\rangle$ in the eigen basis of the Hamiltonian, H=h(1)+λV(2). Then, given a certain observable defined by an operator, O, its expectation value at time *t* is $O\left(t\right)=\langle \Psi (t)|O|\Psi (t)\rangle$. Now, the long time or equilibrium average of the expectation value of O is simply given by ${\langle O\rangle }_{eq}=\sum_{\mu }{\left|C_{\mu }\right|}^{2}\langle E_{\mu }|O|E_{\mu }\rangle$. On the other hand, the statistical average is ${\langle O\rangle }_{stat}=\langle \langle O\rho_{stat}\rangle \rangle$, where $\rho_{stat}$ is the density operator corresponding to an appropriate statistical ensemble. The system is said to be thermalized with respect to the observable O and almost any state, $\left|\Psi (0)\right.\rangle$, if the long time average is the same as the ensemble (statistical) average,

(12) $${\langle O\rangle }_{eq}\approx {\langle O\rangle }_{stat}\;.$$

A practical criterion for testing this is to use the relative error, $\Delta_{0}$, where

(13) $$\Delta_{0}=\left|\frac{{\langle O\rangle }_{eq}-{\langle O\rangle }_{stat}}{{\langle O\rangle }_{stat}}\right|.$$

In order to specify the appropriate statistical ensemble, let us say that the system is prepared in a non-equilibrium state, Ψ(0), with the *H* expectation value in this state, $E_{0}$, and width (square root of the expectation value of $H^{2}-E_{0}^{2}$) ΔE, such that ΔE is much smaller than the spectrum span but large enough to contain many *H* eigenstates. In such a situation, the microcanonical ensemble is the appropriate statistical ensemble. With the energy shell defined by $W\in [E_{0}-\Delta E,E_{0}+\Delta E]$, the microcanonical average is given by

(14) $${\langle O\rangle }_{stat}={\langle O\rangle }_{mic}=\frac{1}{d^{\prime }}\sum_{\mu }^{\prime }\langle E_{\mu }|O|E_{\mu }\rangle ,$$

where $d^{\prime }$ is the number of eigenstates in *W*, and $\sum^{\prime }$ means that the sum is restricted to the eigenstates in *W*.

**Figure 5:**
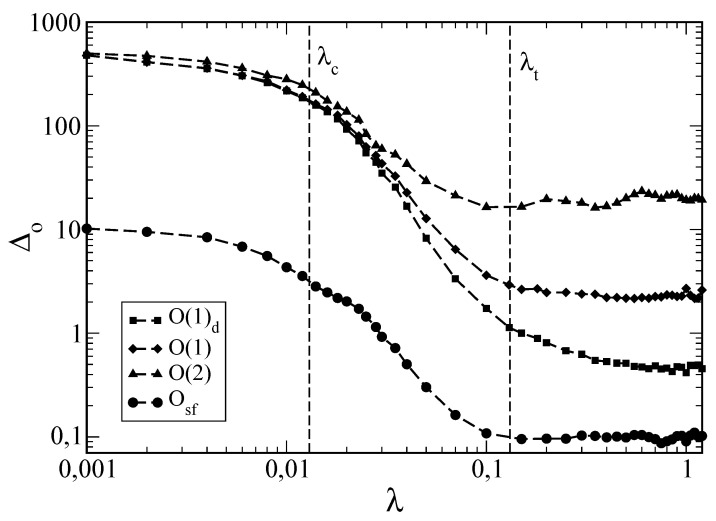
Variation in $\Delta_{o}$ (see Equation (13)) with the interaction strength, λ, for the four operators, O, defined in the text. Results are shown for a 60-member EGOE(1+2) with (m,N)=(6,16), initially prepared in a state, $\Psi^{\left(1\right)}\left(0\right)$. See reference [22] for details of $\Psi^{\left(1\right)}\left(0\right)$. It can be clearly seen that only $O{\left(1\right)}_{d}$ and $O_{sf}$ operators thermalize for $\lambda ∼\lambda_{t}$. For the chaos markers, $\lambda_{c}$ and $\lambda_{t}$, see Section 3.1. Figure is taken from reference [22] with permission from the Institute Of Physics (IOP).

Using three different initial states, four different types of O, three different energy shells and varying λ (strength of the interaction), EGOE(1+2) calculations were performed in reference [22]. For example, the operators used were (i) diagonal one-body operators, $O{\left(1\right)}_{d}=\sum_{k}\theta_{k}a_{k}^{†}a_{k}$; (ii) general one-body operators, $O\left(1\right)=\sum_{k,l}\theta_{kl}a_{k}^{†}a_{l}$; (iii) two-body operators, $O\left(2\right)=\sum_{k<l,p<q}\theta_{klpq}a_{k}^{†}a_{l}^{†}a_{q}a_{p}$; and (iv) strength function operators, $O_{sf}=O^{†}\left(1\right)O\left(1\right)$. It was seen from the results that the onset of GOE spectral fluctuations is not sufficient to guarantee thermalization in finite systems (i.e., $\lambda ∼\lambda_{c}$ will not lead to thermalization). Only when there is quasi-complete delocalization of eigenstates with $\lambda ∼\lambda_{t}$, is there thermalization. More significantly, it was seen that ETH is the mechanism for thermalization with only certain types of observables, such as the strength function (sum rule) operators, i.e., only operators of the type $X^{†}X$ (occupancy operators belong to this class) or sums of such operators will thermalize. As seen clearly from Figure 5, not all types of operators will thermalize. Also, there is clear dependence on the structure of the initial state. Moreover, the typical value of the relative error between the equilibrium and microcanonical averages, $\Delta_{0}^{typ}$, for $X^{†}X$ type operators, is inversely proportional to the square root of NPC in the transition strengths generated by the operator, *X*, (see reference [5] for the definition of NPC in transition strengths) acting on the middle of the microcanonical shell, and this implies that only chaotic systems thermalize. For detail, see reference [22]. Although the numerical results are suggestive that EGOE(1+2) for $\lambda ∼\lambda_{t}$ generates thermalization with the ergodicity principle satisfied, it is important to also produce numerical results for boson systems (using BEGOE(1+2)), fermions with a spin degree of freedom (using EGOE(1+2)-*s* as analyzed in reference [19]) and so on. However, at present, there is a major gap in the analytical understanding of ETH generated by EGOE(1+2). Here, the attempts made in [50] may be useful.

## 4. Random Matrix Analysis of Weakly Interacting Trapped Bosons and Related Systems

Atoms, nuclei and mesoscopic devices of condensed matter systems have been investigated in considerable detail in the past using RMT. Also, as mentioned in Section 2, there are now applications for quantum back holes using the SYK model. Going beyond these, in particular, in probing various aspects of thermalization of isolated finite quantum systems, there is great interest in studying the statistical properties of weakly-interacting trapped bosons (and related systems). These systems belong to the subject of Bose–Einstein condensation (BEC). As already mentioned, experiments with ultra-cold atoms in which the interaction strength and other parameters are controlled provide the main motivation for these studies [61,62,63,64,65,80]. There is now a series of theoretical investigations by a Kolkata group (references [25,26,27,28,29,81,82,83]), and the results of these studies are briefly described in this section. These include (i) an analysis of level fluctuations using GOE and related ensembles in trapped interacting boson systems, diffuse van der Walls clusters and molecular resonances in erbium isotopes; and (ii) statistical relaxation in interaction quench dynamics of ultra-cold bosons and thermalization in isolated quantum systems. Before turning to these, let us add that there have also been important studies on cold atoms using RMT by the Heidelberg group [84,85,86,87].

### 4.1. Results for Spectral Statistics Using GOE and Related Ensembles

#### 4.1.1. Weakly-Interacting Trapped Boson Systems

The energy spectrum of weakly-interacting trapped boson systems is interesting, as it shows a transition from a collective to single particle nature with increasing excitation energy. Using an *ab initio*, but approximate, many-body technique called the potential harmonic expansion method (PHEM) which keeps all possible two-body correlations as developed by a Kolkata group (see reference [25] for a description of this method), the energy levels of zero-temperature, many-boson systems with a boson number varying from 3 to 5000 are calculated. Here, bosons are assumed to be in a 3D, confined, harmonic potential and weakly interact through a two-body van der Waals potential, given by $V(x_{ij})=\infty$ for $x_{ij}\leq r_{c}$ and $-\left(C_{6}\right)/\left(x_{ij}^{6}\right)$ for $x_{ij}>r_{c}$. In the calculations, the value of $C_{6}$ is taken to be $6.4898\times 10^{-11}$ oscillator units (o.u.) as appropriate for ${}^{87}$Rb atoms in a JILA experiment. Similarly, the value of $r_{c}$ is adjusted such that the *s*-wave scattering length is $a_{s}=2.09\times 10^{-4}$ o.u. Let us add that all the PHEM calculations have been carried out by Barnali Chakrabarti and her group in Kolkata.

**Figure 6:**
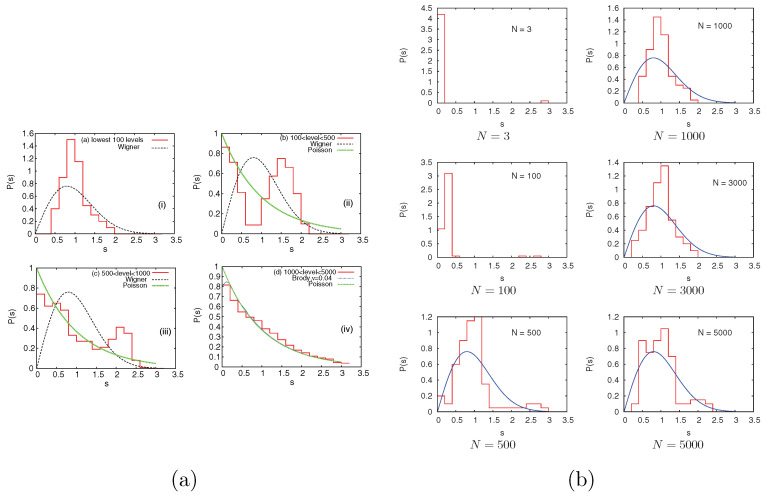
(**a**) Nearest neighbor spacing distribution (NNSD), P(s) vs. *s*, for the trapped bosonsystem defined in the text (histograms) compared to the Wigner (GOE) distribution (in three figures) and Poisson distribution (in the fourth figure) for four segments of the spectrum. The range of levels in each segment is given in the figures. The number of bosons in the calculations is 5000. In the fourth figure, a comparison is also made with the Brody distribution, and the value of the Brody parameter, ν, is given in the figure. (**b**) The NNSD for the same system as in (**a**) but for six different boson number values (in the figures, the boson number is denoted by *N*). The figures in (**a**) are from reference [25] and in (**b**), from reference [26], and they were taken with permission from the American Physical Society.

The calculated energy levels showed that for repulsive BEC, there is a transition from the Wigner-like form, displaying a level repulsion to Poisson distribution in the nearest neighbor spacing distribution (NNSD) with an increase in excitation energy (see Equation (15) ahead for the Wigner and Poisson forms). For repulsive interactions, the lower levels are correlated and manifest level repulsion. For intermediate levels, NNSD shows a mixed form which clearly signifies the existence of two energy scales (external trap and inter-atomic interactions). For very high levels, the trapping potential dominates, generating a Poisson distribution. Figure 6a shows these results for 5000 interacting bosons (see [25] for detail). Similarly, the power spectrum of the energy levels showed $1/f^{\alpha }$ noise with 1≤α≤2, and this is one of the few known realistic examples showing this feature [26]. For example, for 5000 bosons, the α value for the lowest 500 levels is 1.3, for 500–1000 levels, it is 1.72 and for 1000–5000 levels, it is 1.99 (note that α=2 for Poisson and 1 for GOE). These results are consistent with the observation in Figure 6a. Besides a varying number of levels, for a fixed number of low-lying levels by varying number of bosons, it is seen from Figure 6b that for small boson systems, the spacing distribution shows a Shrielman peak which arises due to the large number of quasi-degenerate states (besides this, the NNSD and number variance exhibit deviations from Poisson statistics similar to those of a rectangular billiard [81]). Also, as seen from Figure 6b, with an increasing boson number, the lower levels in the system change smoothly from Poisson to Wigner-like. Finally, measures for higher-order correlations in spectral fluctuations are also studied. For 5000 bosons and focusing on the lowest 100 levels, the *q*-th order correlation function $C_{q}\left(n\right)$ was calculated for 1<q≤10 using a two-fold averaging [82]. The numerical results clearly show that the level fluctuations follow GOE random matrix predictions, even for higher orders, i.e., a logarithmic correlation structure is seen instead of a multi-scaling structure in the $C_{q}\left(n\right)$ results. All these results are significant, as experimental studies of interacting trapped boson systems are possible.

#### 4.1.2. Diffuse van der Waals Clusters

The statistical properties of eigen energies, given by NNSD P(s), the level number variance, $\Sigma^{2}\left(L\right)$, and the Dyson–Mehta $\Delta_{3}\left(L\right)$ statistic, were calculated in reference [27] for diffuse van der Waals clusters of different sizes, i.e., three-dimensional, ultra-cold, bosonic clusters with, say, *N* Rb atoms, with *N* varying from 3 to 40, and interacting through a two-body van der Waals potential. Let us add that the experimental realization of van der Waals bosonic clusters is possible [88]. For large clusters, it was found that the level fluctuations are close to those of GOE, although true signatures of quantum chaos were not seen. However, the contrasting conjecture of Berry and Tabor (i.e., Poisson statistics) was observed with a smaller cluster size. For small clusters, due to the existence of a large number of quasi-degenerate states in the low-lying part of the spectrum, a Shnirelman peak in P(s) distribution was seen. It was also found that there is a narrow region of intermediate spectra which can be described by semi-Poisson statistics (see Equation (16) ahead), whereas the higher levels are regular and exhibit Poisson statistics. These observations were further supported by an analysis of the distribution of the ratio of consecutive level spacings P(r) which is independent of the unfolding procedure (See Equation (18) ahead) and thereby, provides a tool for more transparent comparison with experimental findings than P(s).

#### 4.1.3. Molecular Resonances in Erbium Isotopes

Recently, Frisch et al. [89] studied collisions of trapped cold Erbium atoms, ${}^{166}$Er and ${}^{168}$Er isotopes, as a function of the magnetic field (*B*) and observed many Feno–Feshbach resonances. This is the first and the only experimental demonstration of quantum chaos (RMT) at ultra-cold temperatures. Frisch et al. stated that “given the complexity of the scattering, the analysis of ultra-cold collision data cannot and should not aim anymore at the assignment of individual resonances”. They statistically analysed the resonances using RMT and found intermediate statistics between Poisson and GOE. Later, a reanalysis of the same spectra was done by Mur-Petit and Molina [90] from the view point of missing resonances using RMT and concluded that the disagreement with GOE is due to the possibility of missing resonances (about 20%) in the spectrum. Going beyond these analyses, Roy et al. [83] employed a large number of measures for level fluctuations and concluded from their thorough analysis that the resonances exhibit semi-Poisson characteristics. Here, below, we describe this in some detail.

In reference [83] the NNSD P(s), ratio of nearest spacings P(r), $\Delta^{2}$ test measuring the distance between the numerical data and theoretical predictions and the power spectrum $P_{k}^{\delta }$ were analysed. For the NNSD, the P(s) for the well known Poisson (*P*), Wigner (*W*) or GOE were used:

(15) $$P_{P}\left(s\right)=\exp\left(-s\right)\;,\;\;\;P_{W}\left(s\right)=\left(\pi s/2\right)\;\exp\left(-\pi s^{2}/4\right)\;,$$

as well as the general semi-Poisson, $P_{\nu }\left(s\right)$, with ν=1, giving the semi-Poisson (*SP*) [91],

(16) $$P_{\nu }\left(s\right)=\frac{{\left(\nu +1\right)}^{\nu +1}}{\Gamma (\nu +1)}s^{\nu }\;\exp\left(-\left(\nu +1\right)s\right)\;,\;\;\;P_{\nu =1}\left(s\right)=P_{SP}\left(s\right)=4s\exp\left(-2s\right)\;,$$

and an NNSD, P(s,f) [92], appropriate for the superposition of resonances with different symmetries (f=0 Poisson, f=1 W, f=0.72 for $P_{SP}\left(s\right)$),

(17) $$P\left(s,f\right)=\left(1-f+\frac{\pi }{2}Q\left(f\right)s\right)\;\exp\left(-\left(1-f\right)s+\frac{\pi }{4}Q\left(f\right)s^{2}\right)\;;\;\;\;Q\left(f\right)=0.7f+0.3f^{2}\;.$$

**Figure 7:**
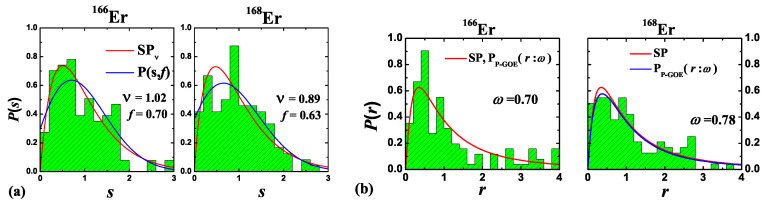
(**a**) NNSD P(s) vs. *s* for ${}^{166}$Er and ${}^{168}$Er resonances (histograms) compared with $P_{SP}\left(s\right)$ and P(s,f) distributions (smooth curves). (**b**) for the same data but for the ratio of nearest spacings distribution P(r) vs. *r* (histograms) compared with $P_{SP}\left(r\right)$ and $P_{P-GOE}$(*r*:ω) distributions (continuous curves). Note that the P(r) curves for $P_{SP}\left(r\right)$ and $P_{P-GOE}$(*r*:ω) are indistinguishable for ${}^{166}$Er. The data used for the figure is from the work reported in reference [83].

A comparison of the NNSD constructed after unfolding the spectra, from the observed resonances (treating them as levels) with the P(s) distributions showed clearly that the SP fitted best. Also, the deduced Brody parameter (for the Poisson to W transition) value was 0.7 for ${}^{166}$Er and 0.78 for ${}^{168}$Er, while its value for $P_{SP}\left(s\right)$ was ∼0.7. Similarly the fit to $P_{\nu }$ gave ν=1.02 and 0.89, respectively. Also, the deduced *f* values in P(s,f) were 0.7 and 0.63, respectively, and its value for $P_{SP}$ was 0.72. In addition, the P(s) from a 2×2 random matrix interpolating Poisson and Gaussian [93] also showed that $P_{SP}$ gave the best representation of the data. See Figure 7a, and, for more detail, reference [83].

In order to eliminate the dependence on unfolding, the distribution of the ratio of nearest spacings given by [94] is also used:

(18) $$P_{P-GOE}\left(r:\omega \right)=\frac{1}{Z_{\omega }}\;\frac{{\left(r+r^{2}\right)}^{\omega }}{{\left(1+\left(2-\omega \right)r+r^{2}\right)}^{1+\frac{3}{2}\omega }},$$

where ω=0 gives Poisson, ω=1 W (GOE) and ω∼0.7 for $P_{SP}$. The $Z_{\omega }$ in Equation (18) is a normalization constant. As seen from Figure 7b, the agreement with $P_{SP}$ is good. Finally, the power spectrum analysis is also consistent with SP. Note that the power spectrum shows $1/f^{\alpha }$ noise with α=2 for Poisson, α=1 for GOE and α∼1.7 for SP. The data gives α=1.51±0.15 for ${}^{166}$Er and 1.5±0.15 for ${}^{168}$Er. In conclusion, as stated in reference [83], “increasing the magnetic field resolution and producing higher quality data will clearly confirm if there are missing resonances, and a negative result of such an experiment will prove that the resonances are of semi-Poisson character”.

### 4.2. Time Evolution, Quantum Chaos and Decoherence in Ultra-Cold Boson Systems

Going beyond level statistics, entropy production with time and statistical relaxation and their relations to decoherence for a system of 10 bosons in a one-dimensional harmonic trap quenched by a two-body interaction was also studied in reference [29]. Starting with the time dependent Schrödinger equation, $\hat{H}\Psi =i\frac{\partial \Psi }{\partial t}$, and taking the Hamiltonian as

(19) $$\hat{H}\left(x_{1},x_{2},\ldots ,x_{m}\right)=\sum_{i=1}^{m}\hat{h}\left(x_{i}\right)+\Theta \left(t\right)\sum_{i<j=1}^{m}\hat{W}\left(x_{i}-x_{j}\right)\;,$$

the time evolution of a system of m=10 identical bosons in N=6 sp states was studied using the multiconfigurational time-dependent Hartree for bosons (MCTDHB) developed by the Heidelberg group [95,96,97]. In Equation (19), $\hat{h}\left(x\right)=\hat{T}\left(x\right)+\hat{V}\left(x\right)$ is the one-body Hamiltonian in the external trapping potential, $\hat{V}=\frac{1}{2}x_{i}^{2}$, and the kinetic energy, $\hat{T}=-\frac{1}{2}{\hat{\partial }}_{x}^{2}$. Similarly, $\hat{W}\left(x_{i}-x_{j}\right)$ is the two-body interaction and Θ(t) is the Heaviside function. Since the time-evolution starts at t=0, the Θ(t) term in Equation (19) implements the interaction quench. In the calculations, a contact interaction with a strength of λ is considered, i.e., $\hat{W}\left(x_{i}-x_{j}\right)=\lambda \delta \left(x_{i}-x_{j}\right)$. Similarly, the many-body wave functions are constructed as

(20) $$|\psi \left(t\right)\rangle =\sum_{\vec{n}}C_{\vec{n}}\left(t\right)|\vec{n};t\rangle ,$$

with the summation running over all possible N+m−1m configurations $|\vec{n};t\rangle =|n_{1},\ldots ,n_{N};t\rangle$, $\sum_{i}n_{i}\equiv m$. Note that both the expansion coefficients and the sp states in Equation (20) are time-dependent. Let us add that the MCTDHB calculations were done by Axel Lode and Barnali Chakrabarti.

**Figure 8:**
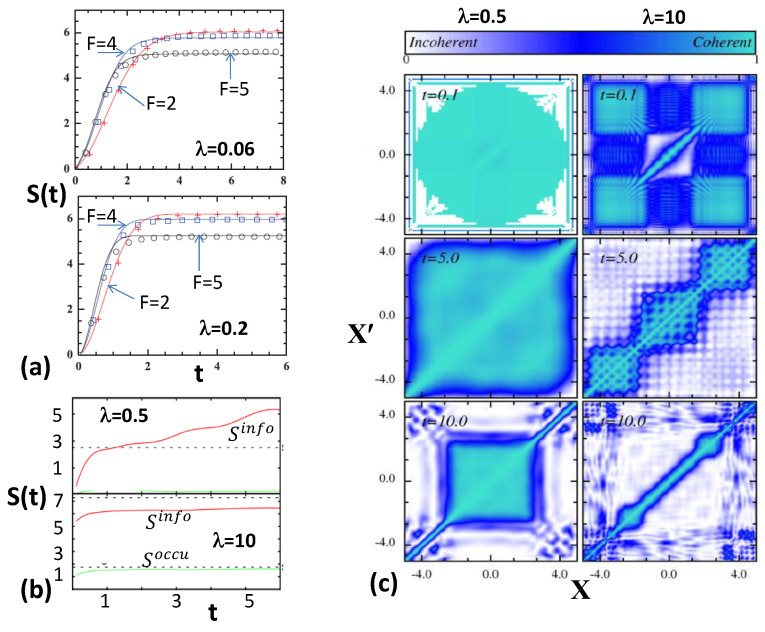
(**a**) Entropy, S(t), defined by the return probabilities vs. time *t* for a BEGOE(1+2)-*F* ensemble for two different values of the interaction strength, λ=0.06 (this gives the BW to Gaussian transition region) and λ=0.2 (this gives the Gaussian region). The histograms are ensemble results, and the smooth curves are from EE theory. See reference [21] for detail, and the figures are taken from this paper with permission from the Institute of Physics. (**b**) Many-body Shannon or information entropy $S^{info}\left(t\right)$ (red curves) and occupational entropy $S^{occu}\left(t\right)$ (green curves) for different inter-particle interaction strengths for the trapped boson system with interaction quench described in the text. Dashed lines are GOE curves. See text for further details. (**c**) The first-order correlation function, $g(x,x^{\prime };t)$, for the system described in the text as a function of the interaction strength, λ and time *t*. The left column represents λ=0.5 (weak) and the right column represents λ=10 (strong). The results are shown for t=0.1, 5 and 10. Figures (**b**) and (**c**) are taken from reference [29] with permission from the American Physical Society.

Using the coefficients, $C_{\vec{n}}\left(t\right)$, both the information entropy, $S^{info}$, and occupation entropy, $S^{occu}$, were examined as functions of time, *t*, and interaction strength. It was seen that for small values of interaction strength, λ (calculations used λ=0.5), entropies increase with time and do not saturate, while for large interaction strengths (λ=10), entropies increase with time initially, and after some time, they are found to saturate, as shown in Figure 8b. The entropy increase and saturation with time is also a feature of EE, as seen from Figure 8a. The saturation value for $S^{info}$ for the trapped boson system shows clear deviation from the GOE value (EE predicts departure from GOE [5]). Note that the saturation of $S^{info}$ and $S^{occu}$ imply statistical relaxation. More importantly, the production of entropy is closely connected to the buildup of correlations and loss of coherence (or generation of decoherence). Towards this, the first order correlation function is investigated

(21) $$g^{\left(1\right)}\left(x_{1}^{\prime },x_{1};t\right)=\frac{\rho^{\left(1\right)}\left(x_{1}|x_{1}^{\prime };t\right)}{\sqrt{\rho \left(x_{1},t\right)\rho \left(x_{1}^{\prime },t\right)}},$$

where ρ is the diagonal part of the one-body density matrix, $\rho^{\left(1\right)}$, which is given by

(22) $$\rho^{\left(1\right)}\left(x_{1}|x_{1}^{\prime };t\right)=\left(m\right)\int \psi^{*}\left(x_{1}^{\prime },x_{2},\ldots .x_{m};t\right)\psi \left(x_{1},x_{2},\ldots .x_{m};t\right)dx_{2}dx_{3}\ldots .dx_{m}\;.$$

In Figure 8c, results for $g^{\left(1\right)}$ are shown for λ=0.5 and 10 and also for three values of time, *t*. It can be clearly seen from this figure that for large interaction strengths, and after a sufficiently long time, there is decoherence. Let us add that $|g^{\left(1\right)}|$ quantifies the coherence and fringe visibility in interference experiments. $|g^{\left(1\right)}|<1$ means that the fringes in interference experiments will be less than 100%, and thus, there is loss of coherence; $|g^{\left(1\right)}|=1$ corresponds to full coherence. Clearly, the stronger the inter-particle repulsion, the stronger the loss of coherence. Finally, there is a strong link between the dynamics of entropy and decoherence as a large production of entropy causes an intensified loss of coherence, implying statistical relaxation, and most importantly, decoherence via fringe visibility in interference experiments can be measured experimentally.

Besides the results for a realistic isolated interacting boson system described above, second-order coherence is also examined using the second-order correlation function:

(23) $$g^{\left(2\right)}\left(x_{1},x_{2};t\right)=\frac{\rho^{\left(2\right)}\left(x_{1},x_{2};t\right)}{\rho \left(x_{1};t\right)\rho \left(x_{2};t\right)},$$

where $\rho^{\left(2\right)}$ is the diagonal part of two-body reduced density matrix. The $g^{\left(2\right)}$ measures how much the simultaneous measurement of two bosons at $x_{1}$ and $x_{2}$ differ from the separate measurement of two bosons at $x_{1}$ and $x_{2}$. From numerical calculations [28], it is seen that strongly-interacting, isolated quantum, many-body systems will thermalize with the clear signature of saturation in entropy. Also, coherence propagates with time, with a quick loss in first-order coherence, and develop the anti-bunching effect in second-order coherence. These observations are fundamentally related to the delocalized and fragmented many-body state. However, the weakly-interacting many-body state, which is localized and in the condensed phase, will exhibit fluctuations in the dynamics of entropy and retain both first-order and second-order coherence with time and never thermalize. In addition, more interestingly, there was an attempt recently to study decoherence using EE [51]. In this preliminary attempt, decoherence of a quantum bit interacting with an environment modeled by an embedded ensemble for fermions and bosons was studied. The numerical results showed the dependence of decoherence on the nature of the environment.

## 5. Conclusions

Embedded *k*-body ensembles that are paradigmatic models for many-body chaos and thermalization in isolated finite quantum (fermion or boson) systems were briefly reviewed with emphasis on recent results and applications. Firstly, (in Section 2) embedded ensembles with Lie algebraic symmetries for fermion and boson systems that have been studied in the literature and their possible extensions for Majorana fermions, with point group symmetries and so on, were discussed. Secondly, (in Section 3) the results generated by these ensembles for various aspects of chaos, thermalization and statistical relaxation, including the role of *q*-Hermite polynomials in these ensembles, were presented. Finally, (in Section 4) very recent analyses of numerical and experimental data for level fluctuations in trapped boson systems and results for statistical relaxation and decoherence in these systems with close relations to results from embedded ensembles were presented. The construction and analysis of new embedded ensembles, more detailed applications for various aspects of thermalization in isolated quantum systems, the development of new analytical methods so that two-point and higher-point correlations generated by EE can be understood, more detailed analyses of trapped boson systems (for example, with long range interactions, spinor BEC etc.) and further exploration of the role of the generating functions of *q*-Hermite polynomials in not only one variable, but also in two or more variables (these will be useful in the study of transition strengths [5] and see reference [98] for *q*-multivariate Gaussians) are some of the new directions. More importantly, experimental tests of the predictions of EE, say, using cold atoms, are essential for firmly establishing EE in quantum physics. Here, for example, experimental determination of the transition markers $\lambda_{c}$, $\lambda_{F}$ and $\lambda_{t}$ in realistic systems, such as trapped boson systems, will be important.
